# Elevated Concentration of Defensins in Hepatitis C Virus-Infected Patients

**DOI:** 10.1155/2016/8373819

**Published:** 2016-06-20

**Authors:** Ehab H. Mattar, Hussein A. Almehdar, Abdullah A. AlJaddawi, Isam ElDin M. Abu Zeid, Elrashdy M. Redwan

**Affiliations:** ^1^Department of Biological Sciences, Faculty of Sciences, King Abdulaziz University, P.O. Box 80203, Jeddah, Saudi Arabia; ^2^Therapeutic and Protective Proteins Laboratory, Protein Research Department, Genetic Engineering and Biotechnology Research Institute, City for Scientific Research and Technology Applications, New Borg EL-Arab, Alexandria 21934, Egypt

## Abstract

Hepatitis C virus (HCV) is the major etiological agent of human non-A and non-B hepatitis, affecting around 180 million people worldwide. Defensins, small cysteine-rich cationic peptides, are shown to have potent antibacterial, antiviral, and antifungal properties. Defensins can be found in both normal and microbial infected patients, at variable concentrations. Notably, viral infections are often associated with elevated concentrations of defensins. The current study aimed to estimate the concentrations of total, *α*-, and *β*-defensins in serum taken from normal and HCV-infected patients. 12 healthy (noninfected) and 34 HCV-infected patients were enrolled. Standardized immunoassay kits were used to obtain serum concentrations of defensins. The obtained results were calibrated against kit standard reagents. Total defensin concentrations in HCV-infected patients were significantly higher (2- to 105-fold) compared to healthy individuals. The concentrations of *α*-defensins were also significantly elevated in the HCV-infected patients (31–1398 ng/50 *μ*L). However, concentrations of *β*-defensins ranged from 44.5 ng/50 *μ*L to 1056 ng/50 *μ*L. The results did not reveal differences in serum defensin concentration between male and female HCV-infected patients. A-defensin concentration of ≥250 ng/50 *μ*L was found to contain more *β*-defensins than total defensins and *α*-defensins. This study concludes, for the first time, that serum defensin levels are elevated in HCV-infected patients.

## 1. Introduction

Hepatitis C virus (HCV) is an enveloped, single positive-stranded RNA virus that belongs to the* Flaviviridae* family. Its genome consists of around 10,000 nucleotides and encodes a single polyprotein of 3010–3033 amino acids. HCV polyprotein is cleaved by both host cell and viral proteases into at least 10 distinct structural and nonstructural protein products. The major structural proteins are a core (C) protein, two envelope proteins, E1 and E2, and a short hydrophobic peptide p7 [1]. HCV is a major cause of parenterally transmitted non-A and non-B hepatitis worldwide [2], and infection with HCV is one of the leading causes of chronic liver disease worldwide [3, 4]. The prevalence of HCV infection has increased during recent years; it is estimated that over 180 million people worldwide are infected with HCV, representing 3% of the world's population, while, in countries like Egypt, the incidence of HCV infection is as high as 15% [5]. Efforts to achieve a breakthrough in antiviral clinical research for chronic HCV are currently underway in Western countries [6] and Japan [7].

Today, there is no available vaccine against HCV, and the current treatment for HCV infection is limited to nonselective alpha-interferon (IFN) and ribavirin. However, the Food and Drug Administration has recently approved a list of novel anti-HCV compounds [8]. These emerging antivirals should increase treatment options, particularly for difficult-to-treat patients, such as those suffering from advanced liver diseases or other coinfections, who have poor response rates to current regimens. Although the currently clinically approved and cocktail of anti-HCV therapy is believed to cure more than 90% of infected patients, incidence of viral resistance, null responders, and treatment failure (in addition to poor side-effect profiles and large treatment costs) poses a major limitation that must be resolved. As an RNA virus, HCV very easily develops a resistance to antiviral treatments, due to its error-prone replication properties. Most entry inhibitors (a class of antiretroviral agents) target the host cell components required for HCV entry, such as receptors or key enzymes, and have high genetic barriers to resistance due to their conserved nature. Therefore, these inhibitors tend to not only have pan-genotypic activity against viral infection but also have a greater risk of simultaneously causing cellular toxicity [9].

Defensins belong to a diverse group of antimicrobial peptides with pronounced antimicrobial activity [10–17]. These are short, cationic cysteine-rich polypeptides, which are well known for their high and broad antimicrobial properties [16, 17]. Originally isolated from human and rabbit neutrophils (the most abundant type of white blood cells in most mammals, accounting for 40–75% of white blood cells) [18], defensins have also been found in various other vertebrates [19], invertebrates [20], insects [21], and plants [22, 23]. These polypeptides play important roles in innate immunity against microbial and viral infections, are involved in adaptive immunity, and are also involved in inflammation, wound repair, expression of cytokines and chemokines, production of histamine, and enhancement of antibody responses [23–26]. They are also able to induce and augment antitumour immunity when fused with the nonimmunogenic tumour antigens [27]. Defensins are also activated in signal transduction and regulation of the inflammatory effects, participate in wound healing and chemotaxis, control proliferation, and regulate the release of cytokines [28–30]. Defensin concentrations are shown to be elevated following microbial infection [31]. Levels of defensins in HCV-infected patient have not been estimated yet, which was the main aim of current study.

## 2. Materials and Methods

### 2.1. Samples and Defensin Estimation Kits

HCV-infected patients (34 samples: 19 male and 15 female) and healthy volunteers (12 samples: 6 male and 6 female) participated in this study. The enrolment criteria were based on thorough history taking: patients were considered eligible if (1) no coinfection with HIV or hepatitis B virus (HBV) was present; (2) they suffered from HCV disease and underwent a complete clinical and laboratory evaluation, including tests for liver function; and (3) their serum contained HCV antibodies (confirmed by measuring serum HCV-RNA titre using quantitative real-time polymerase chain reaction (RT-PCR) and TaqMan technology) [32]. Only HCV genotype 4a-infected patients were enrolled in this study. Venous blood samples were collected from all participants. Blood samples were set to clot and sera were separated by centrifugation, collected, aliquoted, and then stored at −80°C prior to use. Finally, all subjects were informed of the aims of the study and oral consent to participation was given. The study protocol was approved by the local ethical committee and conformed to the ethical guidelines of the 1975 Declaration of Helsinki. Total, *α*-, and *β*-defensin estimation kits were purchased from MyBioSource (San Diego, California, USA).

### 2.2. Defensin Concentration Estimation Kits

Human total, *α*-, and *β*-defensin concentration estimation kits were used in accordance with the manufacturer's instructions (MyBioSource, San Diego, California, USA) and standard laboratory enzyme-linked immunosorbent assay (ELISA) protocol, as previously described [33–36]. In brief, 50 *μ*L infected or noninfected serum samples, as well as the standard reagent, were pipette into microelisa strips (in duplicate) and incubated at 37°C for 60 min, followed by a wash with washing solution (3x). 100 *μ*L horseradish peroxidase (HRP) reagent was added to each well and incubated for 60 min at 37°C, followed by wash with washing solution (4x). 50 *μ*L of chromogen A and 50 *μ*L chromogen B were added to each strip well, then gently mixed, and incubated for 15 min at 37°C, away from direct light, following which 50 *μ*L stop solution was added to each well. Following an observed colour change from blue to yellow, the optical density (OD) was read at 450 nm within 15–30 min after adding the stop solution. The output reading results were calculated, as per manufacturer's instructions (MyBioSource, San Diego, California, USA), as the average of the duplicate readings for each standard and sample by subtracting the average optical density of the blank/control (*V*_B/C_).

### 2.3. Statistical Analysis

Raw OD data was presented as mean ± SD. The data obtained was analysed using the unpaired *t*-test. *P* values of <0.05 were considered to be statistically significant.

## 3. Results

### 3.1. Defensin Concentrations in Noninfected Individuals

**Figure 1:**
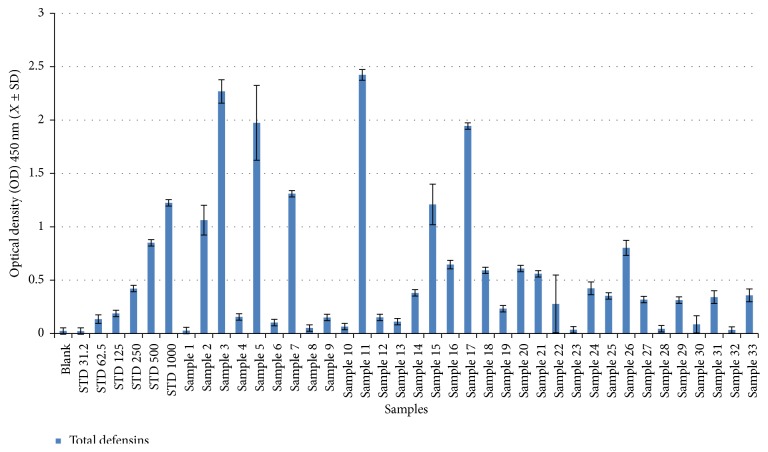
Concentration distribution of human total defensins in patient sera.

**Figure 2 fig2:**
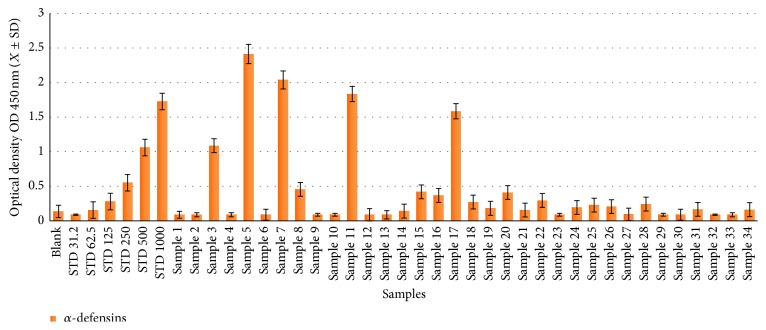
Concentration distribution of human *α*-defensins in patient sera.

**Figure 3 fig3:**
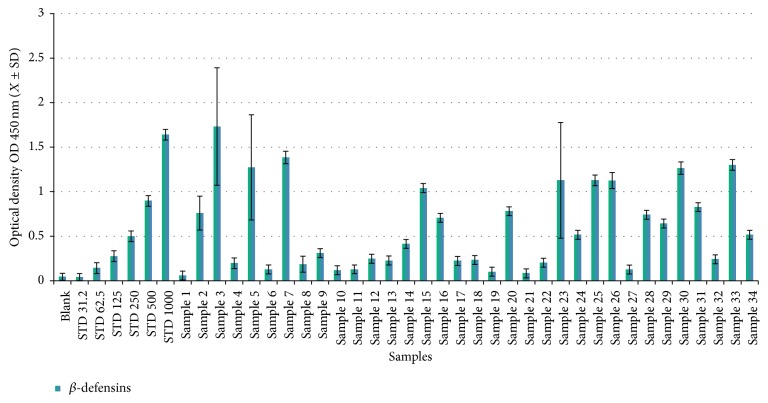
Concentration distribution of human *β*-defensins in patient sera.

**Figure 4:**
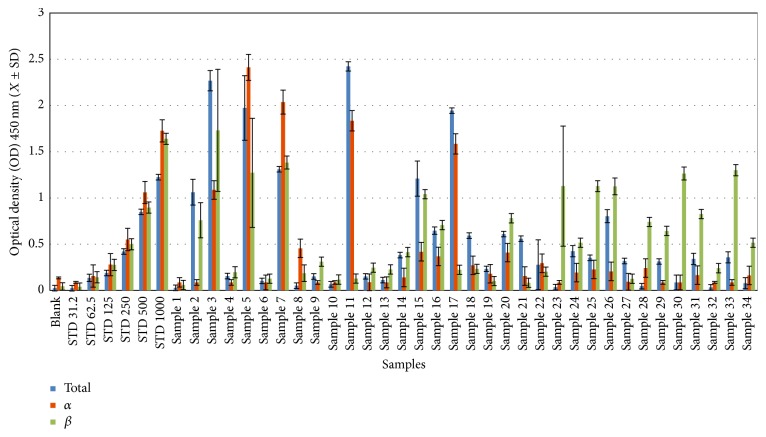
Comparison of concentration distribution of human (total, *α*, and *β*) defensins in patient sera.

In total, 46 human sera were used to calculate human defensin concentrations (total, *α*-, and *β*-defensins) using a commercial ELISA (Section 2.2). The serum concentrations of total, *α*-, and *β*-defensins in noninfected samples ranged from 18.66 to 2.88 ng/50 *μ*L with a mean concentration (±SD) of 11.68 ± 8.1 ng/50 *μ*L (Figures 1 [Fig fig2] [Fig fig3]–4 and Tables 1 [Table tab2] [Table tab3]–4), with no gender-related differences (data not presented). For total defensins, the calculated concentration was 18.66 ± 3.5 ng/*μ*L, corresponding to the kit standard number 1 (31.25 ng/50 *μ*L) and showing a clear significant difference from other kit standards (62.5 to 1000 ng/50 *μ*L). The differences between human *α*-defensin concentrations and the kit standards started from 31.2 ng/50 *μ*L (Figure 1 and Table 1). Human *β*-defensins concentrations were much lower than any kit standards used.

### 3.2. Total Human Defensin Concentrations in HCV-Infected Patients

The serum concentrations of human defensins (total, *α*-, and *β*-defensins) in patients infected with HCV genotype 4a were significantly higher (*P* < 0.0001) compared to control. 5 out of 34 (14.71%) of infected patients had the highest concentrations (1589–1979 ng/50 *μ*L) of total defensins (Figures 1 and 4 and Tables 1 and 4); 3 of the patients were female, without statistical significance. The mean concentrations of total defensins in female patients (449.56 ± 574.53 ng/50 *μ*L) were not different from those in male patients (402.18 ± 562.56 ng/50 *μ*L) (Figures 1 and 4 and Tables 1 and 4).

### 3.3. Human *α*-Defensins Concentrations in HCV-Infected Patients

All HCV-infected patients revealed significantly higher concentrations of human *α*-defensins (31.2–1398 ng/50 *μ*L) in comparison to controls (13.5 ± 1.5 ng/50 *μ*L). The majority of HCV-infected patients (70.8%) showed *α*-defensin concentrations below 250 ng/50 *μ*L, which ranged from 31.2 ng/50 *μ*L to 206.44 ng/50 *μ*L (Figures 2 and 4 and Tables 2 and 4) in their serum. Patients 5, 11, and 17 had the highest *α*-defensin concentrations at 1398.03, 1063.15, and 918.31 ng/50 *μ*L, respectively; two patients (5 and 11) were male and the third patient (17) was female. The concentration of *α*-defensins in both male (218.26 ± 378.55 ng/50 *μ*L) and female (234.29 ± 356.46 ng/50 *μ*L) patients was nearly equal (Figures 2 and 4 and Tables 2 and 4).

### 3.4. Human *β*-Defensins Concentrations in HCV-Infected Patients

Concentrations of *β*-defensins were significantly higher in HCV-infected patient sera (44.50–1056.11 ng/50 *μ*L) compared to controls (2.88 ± 0.14 ng/50 *μ*L). Approximately 50% of the patients showed *β*-defensin concentrations of >250 ng/50 *μ*L, ranging from 258 to 1056 ng/50 *μ*L (Figures 3 and 4 and Tables 3 and 4). The concentrations of *β*-defensins in both male (302.01 ± 280.69 ng/50 *μ*L) and female (370.7 ± 323.8 ng/50 *μ*L) patients were similar (Figures 3 and 4 and Tables 3 and 4). Concentrations of *β*-defensins of 500 ng/50 *μ*L were more commonly found in males (5 patients) than females (3 patients; Table 4). Patients 3, 7, 33, 5, and 30 were found to have highest *β*-defensin concentrations at 1056.11, 843.97, 793.23, 776.15, and 770.97 ng/50 *μ*L, respectively; three of these five patients were female (Figures 3 and 4 and Tables 3 and 4).

## 4. Discussion

Defensins are “magic” 28–42 amino acid cationic peptides, assumed to possess a conserved structural fold containing six highly conserved cysteine residues, which form three pairs of highly conserved intramolecular disulfide bonds [17, 37–40]. Vertebrate defensins are classified as *α*-, *β*-, and *γ*-defensins, based on their cellular origin, the spacing between the cysteine residues, and the number and pattern (topology) of their disulfide bridges [17, 38, 40]. In mammals, barrier epithelial cells mostly generate *β*-defensins, whereas *α*-defensins are mainly stored in the azurophil granules of neutrophils [16]. In the mouse, Paneth cells and fibroblasts produce at least 17 *α*-defensins, whereas various epithelial cells and keratinocytes generate 4 *β*-defensins. The *α*- and *β*-defensins are present in different vertebrate species, where they are found in the granules of immune cells, epithelial tissue, body fluids, and mucosal surfaces [40].

In the current study, circulating concentrations of defensins in HCV patients were evaluated, for first time worldwide, to determine whether levels of defensins altered during HCV infection. Sera from 12 noninfected and 34 HCV-infected patients were harnessed in order to test this, using the commercial ELISA kits. The obtained results demonstrated that HCV-infected patients had significantly increased (*P* < 0.005–0.0001) levels of defensin (total, *α*-, and *β*-defensins) concentrations compared to the noninfected group. The majority of patients (70.8%) revealed *α*-defensin concentrations below 250 ng/50 *μ*L. The concentrations of *α*-defensins in both male (218.26 ± 378.55 ng/50 *μ*L) and female (234.29 ± 356.46 ng/50 *μ*L) patients were virtually equal. The highest *α*-defensin concentrations were reported in patients 5, 11, and 17 (1398.03, 1063.15, and 918.31 ng/50 *μ*L, resp.); two patients (5 and 11) were male and the third patient (17) was female. Approximately half the HCV-infected patients showed *β*-defensin concentrations of >250 ng/50 *μ*L. Concentrations of *β*-defensins of 500 ng/50 *μ*L were more commonly found in males (5 patients) than in females (3 patients). Three of the five HCV-infected patients showing the highest *β*-defensin concentrations were female, although gender did not seem to have a significant effect. The high defensins concentrations within these patients may be due to a comicrobial infection, and/or a patient's infection was in the acute or after acute phase. The latter suggestion may agree with the results of Aceti et al. [41], where a high anamnestic response in defensin concentration was reported after* in vitro* stimulation of PBMCs from chronic HCV-infected patients with HCV proteins (see below).

These results are generally consistent with the only two studies currently available in literature [41, 42]. One report evaluated *α*- and *β*-defensin concentrations in human peripheral blood by measuring mRNA copy number [42], while the second evaluated *α*-defensin mRNA copy number in human peripheral blood mononuclear cells (PBMCs) of patients with chronic HCV infection, after* in vitro* induction with HCV C proteins [41]. Fang et al. [42] concluded that human peripheral blood *β*-defensins 1 and 2 (*DEFB1* and* DEFB2*) genes were transiently expressed following induction with lipopolysaccharide or heat-inactivated bacterial cells, whereas *α*-defensins 1–3 (*DEFA1*–*3*) genes were constitutively transcribed while the *β*-defensin 3 (*DEFB3*) gene was not expressed. The inducible expression of* DEFB1* and* DEFB2* genes displayed interindividual variability; however, the study did not indicate serum concentrations of defensin peptides.

Aceti et al. [41], however, identified and quantified *α*-defensins in PBMCs using mass spectrometry, ELISA, antibacterial activity, and mRNA levels. PBMCs from 3 patients and controls were stimulated with HCV core protein and hepatitis B virus antigen* in vitro* as well as the *α*-defensin mRNAs level was quantified. The authors found that HCV C protein activates transcription of *α*-defensin* in vitro*, and *α*-defensin peptide levels were accordingly significantly increased in patients with chronic hepatitis C (1.103 ± 0.765 ng/10^6^ cells) and chronic hepatitis B (0.53 ± 0.15) compared to healthy controls (0.217 ± 0.09; *P* < 0.001). In patients with chronic hepatitis C, levels of *α*-defensin and antibacterial activity correlated with the liver fibrosis. Aceti et al. [41] suggested that HCV induces *α*-defensin expression and that the high linear correlation of *α*-defensin levels with advancing fibrosis makes measuring these peptides a reliable marker of fibrosis stage.

Higher concentrations of both *α*- and *β*-defensins in our samples may indicate an immune response profile in these patients. The Th-1 immunity profile (IL-2, IL-12, TNF-*α*, and IFN-*γ*) is correlated with liver fibrosis in patients with chronic hepatitis C, whereas Th2 immunity profile (IL-4 and IL-10) cannot control viral clearance [43]. Defensins are considered to be inducers of proinflammatory cytokines (TNF-*α* and IFN-*γ*) and the Th1-skewed immune response [30]. Recently, patients with severe liver fibrosis presented lower frequency of circulating CD8+ T-cells, higher levels of proinflammatory cytokines, lower levels of IL-10, and higher levels of proinflammatory cytokines (TNF and IFN-*γ*) [44], in line with a previous report that found a linear correlation between *α*-defensin levels and advancing liver fibrosis [41].

In a study by Erhart et al. [45], the expressions of various *α*- and *β*-defensins in biopsy samples taken from 35 patients infected with genital (warts) papillomavirus were analysed. The authors found significantly higher expression of *β*-defensin hBD-1 (*P* = 0.03), hBD-2 (*P* < 0.01), and hBD-3 (*P* < 0.001), while *α*-defensins (HNPs 1–3) were scarcely detectable in normal and viral infected tissues [45].

Generally, humans express 6 *α*-defensins and multiple *β*-defensin peptides. A-defensins 1–3 are especially abundant in human neutrophils, constituting 30–50% of the total protein of their azurophil granules [46–48]. Although plasma levels of *α*-defensins normally vary between 40 and 200 ng/mL, higher levels are found at sites of infection, and plasma levels >100 *μ*g/mL may occur during sepsis, intrauterine infections, and bacterial meningitis [47–55]. These plasma *α*-defensin concentrations are higher than those estimated in our study in normal serum, which may reflect the differences in the methods of measurement used.

Increased plasma and bronchoalveolar lavage levels of *α*-defensins have been reported in individuals with* Mycobacterium avium-intracellulare* infection and pulmonary tuberculosis [53, 54]. A-defensin concentrations were not correlated with infection with* Mycobacterium tuberculosis* and/or its multidrug resistant strain [56]. A-defensins are active against a variety of gram-positive and gram-negative bacteria as well as fungi and parasites [47, 57].

Defensins are considered as one of strongest types of central and peripheral defenders, especially in mucosal tissues. They also link the innate and adaptive immunity. There is a close correlation between increased concentrations of *α*- and *β*-defensins in vaginal tissues and fluids following infection. An enormous increase in *α*-defensins and their secretory neutrophils in the vaginal during endometritis was reported [52]. Fan et al. [58] have shown that concentrations of human *β*-defensin 2 and *α*-defensin 5 were increased in women with vaginosis, which the authors considered to be an immune response against bacterial invasion [58]. This was later confirmed [59]; the group showed that bacterial vaginosis was associated with lower vaginal concentrations of *β*-defensin 3, but not *β*-defensin 2 or *α*-defensins 1–3, in pregnant women. Baricelli et al. [60] added that *β*-defensin 2 was secreted in the milk of lactating women. Interestingly, levels of *β*-defensin 2 were found to be significantly higher in colostrum than in mature milk samples [60]. The increased concentrations of various types of defensins in clinical conditions compared to normal health are not limited to microbial infections but extend to different kinds of human diseases [31, 61, 62].

Human natural *α*-defensins (HNPs 1, 2, and 3) concentrations should only be compared with the total amount of HNPs 1–3, measured by radio- and immunoassays. Reportedly, HNPs 1–3 serum concentrations measured with RIA [63] were found to be 250 ng/mL in controls sera, while they were 500 to 1750 ng/mL in patients with various lung diseases. In another study, HNPs 1–3 concentrations in serum were measured with ELISA [64] and revealed as ±7 ng/mL in normal individuals and were significantly increased in colon cancer patients with a median concentration of around 15–29 ng/mL. When comparing the total amount of HNPs 1, 2, and 3, as measured using the developed assay, in serum from IC patients (an average of 1076 ng/mL), these levels complied with the increased concentrations found in the study by Mukae et al. [63], whereas the total measured amounts in the serum batches used for the selectivity assessment (an average of 309 ng/mL) were comparable with the concentrations in healthy controls in the same study [61]. More studies have reported HNPs 1–3 concentrations in plasma: around 200–400 ng/mL in healthy controls, when measured with RIA [31, 53–55, 63, 65, 66], and around 40–100 ng/mL when measured with ELISA [51, 67, 68]. Recent measurements in different plasma batches, used for selectivity assessment, showed between 40 and 175 ng/mL and seem comparable with the concentrations measured using the ELISA method [69].

Since 1997, the worldwide pioneer in the defensins, Lehrer [70], raised fundamental questions regarding defensins concentrations in disease and other related issues. Although a 50 kg female or 70 kg male will produce at least 250–700 mg defensins daily (more when neutrophilic leukocytosis occurs), the low levels of defensins in normal plasma (254.8 pg/mL) account for 0.05% of baseline daily production. Where are the rest of these peptides? Are defensins rapidly degraded, and if so, where does this process occur? Do extracellular defensins leave the circulation as rapidly as they enter it (an easy question, from the standpoint of steady-state kinetics), and how rapidly do they enter it (a more challenging question)? How short is the half-life of plasma defensins and do they recirculate or traverse epithelial barriers? Do extracellular defensins permeate tissues such as the lungs and the gastrointestinal tract? Do they impregnate epithelial cells, basement membranes, and mucosal tissues? All of these crucial questions are yet to be comprehensively and satisfactorily answered.

Although there is a clear correlation between the results presented here and previous reports of human circulated defensin concentrations, the exact concentrations of defensins in health and disease remain to be determined.

To conclude, circulated defensins are measured at significantly higher concentration in HCV-infected patients compared to healthy individuals. Total, *α*-, and *β*-defensin concentrations are all elevated by tenfold in patients with HCV infections.

## Figures and Tables

**Table 1:** Total human defensin concentration in HCV patients serum.

Variables	ANOVA one-way					
	Gender	Mean	SD	*N*	Concentration (ng/50 *μ*L)	*P *value
Blank		0.0145	0.03	4	000	00
Health sample	6 M, 6 F	0.085–0.165		12	18.66 ± 3.5	00
STD 31.2	Standard	0.0235	0.03	2	31.2	00
STD 62.5	Standard	0.1360	0.04	2	62.5	00
STD 125	Standard	0.1885	0.03	2	125	00
STD 250	Standard	0.4210	0.03	2	250	00
STD 500	Standard	0.8500	0.03	2	500	00
STD 1000	Standard	1.2245	0.03	2	1000	00
Sample 1	M	0.0282	0.03	4	37.44	<0.05
Sample 2	M	1.0625	0.14	4	867.701	<0.005
Sample 3	F	2.2685	0.11	4	1852.60	<0.0001
Sample 4	F	0.1562	0.03	4	103.58	<0.05
Sample 5	M	1.9735	0.35	4	1611.68	<0.001
Sample 6	M	0.1032	0.03	4	47.43	<0.05
Sample 7	F	1.3110	0.03	4	1070.64	<0.0001
Sample 8	M	0.0520	0.03	4	69.04	<0.05
Sample 9	M	0.1512	0.03	4	100.30	<0.05
Sample 10	F	0.0655	0.03	4	86.96	<0.05
Sample 11	M	2.4235	0.05	4	1979.180	<0.00001
Sample 12	M	0.1522	0.03	4	100.93	<0.05
Sample 13	F	0.1117	0.03	4	51.33	<0.05
Sample 14	M	0.3817	0.03	4	226.66	<0.005
Sample 15	M	1.2097	0.19	4	987.91	<0.001
Sample 16	F	0.6460	0.04	4	380	<0.005
Sample 17	F	1.9445	0.03	4	1587.99	<0.0001
Sample 18	F	0.5927	0.03	4	242.02	<0.05
Sample 19	M	0.2335	0.03	4	138.7	<0.05
Sample 20	F	0.6095	0.03	4	358.5	<0.005
Sample 21	M	0.5602	0.03	4	329.53	<0.005
Sample 22	M	0.2785	0.27	4	165.4	<0.05
Sample 23	M	0.0360	0.03	4	39.50	<0.05
Sample 24	F	0.4240	0.06	4	251.78	<0.005
Sample 25	F	0.3532	0.03	4	209.74	<0.005
Sample 26	M	0.8032	0.07	4	472.5	<0.005
Sample 27	M	0.3187	0.03	4	189.25	<0.05
Sample 28	M	0.0455	0.03	4	60.41	<0.05
Sample 29	F	0.3135	0.03	4	186.16	<0.05
Sample 30	M	0.0870	0.08	4	115.51	<0.05
Sample 31	M	0.3410	0.06	4	202.5	<0.05
Sample 32	F	0.0340	0.03	4	45.15	<0.05
Sample 33	F	0.3582	0.06	4	212.71	<0.05
Sample 34	F	0.0785	0.06	4	104.22	<0.05

**Table 2 tab2:** Human *α*-defensin concentration in HCV patients serum.

Variables	ANOVA one-way					
	Gender	Mean	SD	*N*	Concentration (ng/50 *μ*L)	*P* value
Blank	0.099		0.09	4	00.00	00
Health sample	6 M, 6 F	0.0442–0.0897		12	13.5 ± 1.2	00
STD 31.2	Standard	0.0870	0.01	2	31.2	00
STD 62.5	Standard	0.1560	0.12	2	62.5	00
STD 125	Standard	0.2790	0.12	2	125	00
STD 250	Standard	0.5510	0.12	2	250	00
STD 500	Standard	1.0600	0.12	2	500	00
STD 1000	Standard	1.7260	0.12	2	1000	00
Sample 1	M	0.0872	0.05	4	31.3	<0.05
Sample 2	M	0.0870	0.03	4	31.2	<0.005
Sample 3	F	1.0872	0.10	4	512.83	<0.005
Sample 4	F	0.0872	0.03	4	31.3	<0.05
Sample 5	M	2.4130	0.14	4	1398.03	<0.0001
Sample 6	M	0.0870	0.08	4	31.2	<0.05
Sample 7	F	2.0375	0.13	4	1180.5	<0.0001
Sample 8	M	0.4550	0.10	4	206.44	<0.05
Sample 9	M	0.0870	0.02	4	31.2	<0.05
Sample 10	F	0.0870	0.02	4	31.2	<0.05
Sample 11	M	1.8350	0.11	4	1063.15	<0.00001
Sample 12	M	0.0880	0.09	4	31.6	<0.05
Sample 13	F	0.0872	0.06	4	31.3	<0.05
Sample 14	M	0.1405	0.10	4	56.3	<0.05
Sample 15	M	0.4190	0.10	4	190.11	<0.05
Sample 16	F	0.3680	0.10	4	164.9	<0.05
Sample 17	F	1.5850	0.11	4	918.31	<0.001
Sample 18	F	0.2720	0.10	4	121.9	<0.05
Sample 19	M	0.1805	0.10	4	72.32	<0.05
Sample 20	F	0.4090	0.10	4	184.21	<0.05
Sample 21	M	0.1555	0.10	4	62.3	<0.05
Sample 22	M	0.2942	0.10	4	131.81	<0.05
Sample 23	M	0.0872	0.02	4	31.3	<0.05
Sample 24	F	0.1930	0.10	4	77.32	<0.005
Sample 25	F	0.2272	0.10	4	101.8	<0.005
Sample 26	M	0.2062	0.10	4	92.4	<0.005
Sample 27	M	0.0937	0.09	4	33.60	<0.05
Sample 28	M	0.2422	0.10	4	108.51	<0.05
Sample 29	F	0.0872	0.02	4	31.3	<0.05
Sample 30	M	0.0872	0.08	4	31.3	<0.05
Sample 31	M	0.1655	0.10	4	66.31	<0.05
Sample 32	F	0.0870	0.01	4	31.2	<0.05
Sample 33	F	0.0872	0.03	4	31.3	<0.05
Sample 34	F	0.1625	0.10	4	65.10	<0.05

**Table 3 tab3:** Human *β*-defensin concentration in HCV patients serum.

Variables	ANOVA one-way					
	Gender	Mean	SD	*N*	Concentration (ng/50 *μ*L)	*P* value
Blank		0.075	0.04	4	00.00	00
Health sample	6 M, 6 F	0.0290–0.0385		12	2.88 ± 0.14	00
STD 31.2	Standard	0.0405	0.04	2	31.2	00
STD 62.5	Standard	0.1425	0.06	2	62.5	00
STD 125	Standard	0.2765	0.06	2	125	00
STD 250	Standard	0.4995	0.06	2	250	00
STD 500	Standard	0.8965	0.06	2	500	00
STD 1000	Standard	1.6395	0.06	2	1000	00
Sample 1	M	0.0590	0.05	4	45.45	<0.05
Sample 2	M	0.7590	0.19	4	379.9	<0.005
Sample 3	F	1.7315	0.66	4	1056.11	<0.0001
Sample 4	F	0.1965	0.06	4	86.2	<0.05
Sample 5	M	1.2725	0.59	4	776.15	<0.001
Sample 6	M	0.1270	0.05	4	55.70	<0.05
Sample 7	F	1.3837	0.07	4	843.97	<0.001
Sample 8	M	0.1860	0.09	4	81.6	<0.05
Sample 9	M	0.3110	0.05	4	155.7	<0.05
Sample 10	F	0.1175	0.05	4	51.53	<0.05
Sample 11	M	0.1285	0.05	4	56.35	<0.05
Sample 12	M	0.2465	0.05	4	111.44	<0.05
Sample 13	F	0.2275	0.05	4	102.8	<0.05
Sample 14	M	0.4145	0.05	4	207.5	<0.05
Sample 15	M	1.0410	0.05	4	634.95	<0.001
Sample 16	F	0.7070	0.05	4	394.31	<0.005
Sample 17	F	0.2235	0.05	4	101.1	<0.0001
Sample 18	F	0.2330	0.05	4	105.33	<0.05
Sample 19	M	0.1015	0.05	4	44.51	<0.05
Sample 20	F	0.7810	0.05	4	390.9	<0.005
Sample 21	M	0.0835	0.05	4	64.33	<0.005
Sample 22	M	0.2030	0.05	4	91.77	<0.05
Sample 23	M	1.1280	0.65	4	688.01	<0.005
Sample 24	F	0.5165	0.05	4	258.0	<0.005
Sample 25	F	1.1275	0.06	4	687.71	<0.005
Sample 26	M	1.1250	0.09	4	686.2	<0.005
Sample 27	M	0.1265	0.05	4	55.5	<0.05
Sample 28	M	0.7410	0.05	4	370.9	<0.005
Sample 29	F	0.6435	0.05	4	322.1	<0.005
Sample 30	M	1.2640	0.07	4	770.97	<0.001
Sample 31	M	0.8270	0.05	4	461.3	<0.005
Sample 32	F	0.2410	0.05	4	108.95	<0.05
Sample 33	F	1.3005	0.06	4	793.23	<0.001
Sample 34	F	0.5160	0.05	4	258.26	<0.005

**Table 4:** Comparison between total, *α*-, and *β*-defensin concentration level in HCV patients serum.

Variables	Concentration (ng/50 *μ*L)				
	Gender	*N*	Total defensin	*α*-defensin	*β*-defensin
Health sample	6 M, 6 F	12	18.66 ± 3.5	13.5 ± 1.2	2.88 ± 0.14
STD 31.2	Standard	2	31.2	31.2	31.2
STD 62.5	Standard	2	62.5	62.5	62.5
STD 125	Standard	2	125	125	125
STD 250	Standard	2	250	250	250
STD 500	Standard	2	500	500	500
STD 1000	Standard	2	1000	1000	1000
Sample 1	M	4	37.44	31.3	45.45
Sample 2	M	4	867.701	31.2	379.9
Sample 3	F	4	1852.60	512.83	1056.11
Sample 4	F	4	103.58	31.3	86.2
Sample 5	M	4	1611.68	1398.03	776.15
Sample 6	M	4	47.43	31.2	55.70
Sample 7	F	4	1070.64	1180.5	843.97
Sample 8	M	4	69.04	206.44	81.6
Sample 9	M	4	100.30	31.2	155.7
Sample 10	F	4	86.96	31.2	51.53
Sample 11	M	4	1979.180	1063.15	56.35
Sample 12	M	4	100.93	31.6	111.44
Sample 13	F	4	51.33	31.3	102.8
Sample 14	M	4	226.66	56.3	207.5
Sample 15	M	4	987.91	190.11	634.95
Sample 16	F	4	380	164.9	394.31
Sample 17	F	4	1587.99	918.31	101.1
Sample 18	F	4	242.02	121.9	105.33
Sample 19	M	4	138.7	72.32	44.51
Sample 20	F	4	358.5	184.21	390.9
Sample 21	M	4	329.53	62.3	64.33
Sample 22	M	4	165.4	131.81	91.77
Sample 23	M	4	39.50	31.3	688.01
Sample 24	F	4	251.78	77.32	258.0
Sample 25	F	4	209.74	101.8	687.71
Sample 26	M	4	472.5	92.4	686.2
Sample 27	M	4	189.25	33.60	55.5
Sample 28	M	4	60.41	108.51	370.9
Sample 29	F	4	186.16	31.3	322.1
Sample 30	M	4	115.51	31.3	770.97
Sample 31	M	4	202.5	66.31	461.3
Sample 32	F	4	45.15	31.2	108.95
Sample 33	F	4	212.71	31.3	793.23
Sample 34	F	4	104.22	65.10	258.26
